# 3'-UTR SIRF: A database for identifying clusters of whort interspersed repeats in 3' untranslated regions

**DOI:** 10.1186/1471-2105-8-274

**Published:** 2007-07-30

**Authors:** Benjamin B Andken, In Lim, Gary Benson, John J Vincent, Matthew T Ferenc, Bianca Heinrich, Larissa A Jarzylo, Heng-Ye Man, James O Deshler

**Affiliations:** 1 Bioinformatics Program, Boston University, Boston, MA, USA; 2 Department of Biology, Boston University, Boston, MA, USA; 3 Department of Computer Science, Boston University, Boston, MA, USA

## Abstract

**Background:**

Short (~5 nucleotides) interspersed repeats regulate several aspects of post-transcriptional gene expression. Previously we developed an algorithm (REPFIND) that assigns P-values to all repeated motifs in a given nucleic acid sequence and reliably identifies clusters of short CAC-containing motifs required for mRNA localization in *Xenopus *oocytes.

**Description:**

In order to facilitate the identification of genes possessing clusters of repeats that regulate post-transcriptional aspects of gene expression in mammalian genes, we used REPFIND to create a database of all repeated motifs in the 3' untranslated regions (UTR) of genes from the Mammalian Gene Collection (MGC). The MGC database includes seven vertebrate species: human, cow, rat, mouse and three non-mammalian vertebrate species. A web-based application was developed to search this database of repeated motifs to generate species-specific lists of genes containing specific classes of repeats in their 3'-UTRs. This computational tool is called 3'-UTR SIRF (**S**hort **I**nterspersed **R**epeat **F**inder), and it reveals that hundreds of human genes contain an abundance of short CAC-rich and CAG-rich repeats in their 3'-UTRs that are similar to those found in mRNAs localized to the neurites of neurons. We tested four candidate mRNAs for localization in rat hippocampal neurons by *in situ *hybridization. Our results show that two candidate CAC-rich (*Syntaxin 1B *and *Tubulin β4*) and two candidate CAG-rich (*Sec61α *and *Syntaxin 1A*) mRNAs are localized to distal neurites, whereas two control mRNAs lacking repeated motifs in their 3'-UTR remain primarily in the cell body.

**Conclusion:**

Computational data generated with 3'-UTR SIRF indicate that hundreds of mammalian genes have an abundance of short CA-containing motifs that may direct mRNA localization in neurons. *In situ *hybridization shows that four candidate mRNAs are localized to distal neurites of cultured hippocampal neurons. These data suggest that short CA-containing motifs may be part of a widely utilized genetic code that regulates mRNA localization in vertebrate cells. The use of 3'-UTR SIRF to search for new classes of motifs that regulate other aspects of gene expression should yield important information in future studies addressing *cis*-regulatory information located in 3'-UTRs.

## Background

Clusters of short interspersed repeats 4–7 nucleotides (nt) in length have been identified as *cis*-elements that regulate several aspects of gene expression. For example, the hexanucleotide motif UGCAUG is repeated 7 times downstream of exon EIIIB in the fibronectin gene, and mutational studies have shown that this motif is required for cell-type specific alternative splicing [1,2]. (A/U)GGG is another repeated motif found in introns that regulates alternative splicing [3]. Translation control can also be regulated by short repeats. The *oskar *gene in *Drosophila*, for example, contains 13 UUUAY motifs interspersed throughout its 3' untranslated region (UTR) that are required for translation of the *oskar *mRNA once it becomes localized to the posterior pole of *Drosophila *oocytes [4].

The localization of specific mRNAs to distinct regions of a cell is another aspect of gene control that involves short repeated motifs. This mechanism of gene regulation is one way that proteins become distributed to subcellular sites where they are needed. Since oocytes and neurons are highly polarized cells, both are extensively used as model systems for studies directed towards understanding the mechanisms of mRNA localization in animals. Interestingly, several proteins, such as Staufen [5-7] and Kinesin 2 [8,9], mediate mRNA localization in oocytes as well as in neurons. This suggests that the *cis*-elements that specify mRNA localization in both oocytes and neurons may also share some common characteristics.

The *cis*-elements that specify mRNA localization are generally found in 3'-UTRs [10-12], and the role of short motifs in mRNA localization [13,14] was initially discovered by visual inspection of the *Vg1 *mRNA localization element (LE) in *Xenopus *[15]. The motif most important for *Vg1 *mRNA localization, UUCAC, is repeated four times in the ~350 nt *Vg1 *LE. Subsequently, UUCAC motifs were discovered to be important for the localization of another *Xenopus *mRNA, *VegT *[16,17]. This motif is bound specifically by the RNA localization factor Vera/Vg1-RBP (called ZBP1 in neurons and fibroblasts[18]) in both the *Vg1 *and the *VegT *LE. Binding of this protein to UUCAC motifs is thought to facilitate the formation of ribonucleoprotein complexes competent for localization [13,17,19].

*Vg1 *and *VegT *mRNAs both localize during mid-oogenesis in *Xenopus*. In a search for candidate motifs that may specify localization during early oogenesis in *Xenopus*, we developed a novel computational algorithm (REPFIND) [20]. REPFIND facilitates the identification of short repeated motifs by assigning a P-value to all repeated motifs in an input nucleotide sequence, thus identifying the most significant repeats of any size. Using this algorithm we discovered UGCAC is an essential motif for RNA localization during early oogenesis. In addition, we showed that clusters of short CAC-containing motifs are a general and evolutionarily conserved *cis*-element for localization of many mRNAs in oocytes throughout the chordate lineage [20]. Moreover, we showed that REPFIND reliably predicts new localized mRNAs from a 3' UTR database compiled from *Xenopus *cDNA sequences obtained from NCBI [9,20].

In an effort to facilitate the discovery and characterization of new localized mRNAs and RNA localization elements in humans and other mammals, we used the REPFIND algorithm to construct a database of all repeated motifs (3–255 nucleotides long) present in the 3'-UTRs of ~60,000 genes from seven vertebrate species. A web search engine was also constructed which enables one to identify genes that contain an abundance of any selected class of repeated motif. Using this tool we identified hundreds of genes with significant clusters of short CAC- and CAG-containing repeats similar to those of known localized mRNAs. Four of these candidate mRNAs were shown by *in situ *hybridization to be localized to the neurites of cultured neurons.

## Construction and content

### Data collection

All DNA sequence data used to generate the 3'-UTR SIRF motif database were obtained using the NCBI MGC retrieval tool located at: . The mammalian gene collection (MGC) contains thousands of high quality cDNA sequences from seven organisms. The zebrafish gene collection (ZGC) and Xenopus gene collections (XGC) are part of the MGC and were included in all database constructions. At the time we extracted the sequence data the following numbers of sequences were retrieved for each organism:

*Bos taurus*: 1523

*Dario rerio*: 7965

*Homo sapiens*: 20924

*Mus musculus*: 16594

*Rattus norvegicus*: 5100

*Xenopus laevis*: 8406

*Xenopus tropicalis*: 2921

The MGC was utilized because each included gene has all of the annotations that are needed to properly extract the 3'-UTR of the gene. Additionally, the genes included in the MGC are estimated to be full-length cDNAs which are most likely to contain entire 3'-UTRs.

A Perl XML parser was written to extract the useful information from each gene.

The data collected from each gene included:

INSDSeq_length

INSDSeq_update-date

INSDSeq_create-date

INSDSeq_primary-accession

INSDSeq_definition

INSDSeq_sequence

INSDSeq_organism

INSDFeature_interval-from

INSDFeature_interval-to

These data were stripped out of the XML files and inserted into a new database that was constructed specifically for the purposes here.

### Randomly Generated Genes

**Figure 1:**
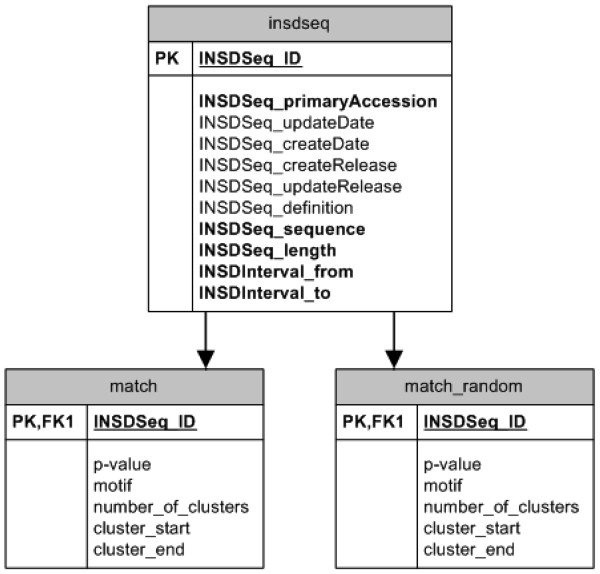
**Schematic representation of the information stored in the 3'-UTR SIRF database**. Sequences were extracted from the Mammalian Gene Collection (NCBI) and stored in the insdseq table of the database. REPFIND was then used to identify clusters of all perfect repeats in the 3'-UTRs of these sequences. The results of this computational analysis were stored in the 'match' table. A similar table, 'match_random' was generated on the same sequences which had their nucleotides shuffled in a random fashion. All information included in the insdseq table is from the NCBI database, except INSDSeq_Create_release, which defines when the table entry was created and INSDSeq_Update_release, which identifies when the table entry is modified. INSDSeq_ID is used as the identification number into the table. It has the same role as INSDSeq_primaryAccession, but is used because it is an integer that is more efficient for indexing. INSDSeq_ID in the match and match_random tables indicates the gene corresponding to the cluster identified by REPFIND. In addition, the P-value, sequence of the repeat (motif), number of motifs, start (cluster_start), and end (cluster_end) of each cluster are shown. These last two entries are used to calculate the size of each identified cluster.

For statistical comparisons, we created a database of 'randomized' 3'-UTRs to be used as a control database generated by REPFIND. To generate a database of an identical number of sequences with identical lengths and nucleotide frequencies, but randomized sequences, each nucleotide in the real 3'-UTR was randomly swapped with another nucleotide in the same 3'-UTR. This was done for each 3' UTR to create a randomized 3' UTR of identical nucleotide composition and length. REPFIND was used to identify repeated motifs in each of the real and shuffled 3'-UTR sequences, and the results were stored in the 'match' and 'match_random' tables, respectively (Figure 1).

### Database Implementation

The database implemented in MySQL is divided into two sections. The first stores the gene sequences obtained from NCBI. The schema for storing the sequences is shown in Figure 1, and the entire gene sequence was stored with exactly one entry per gene in the *INSDSeq Table*. This table uses a binary tree index since the data are read only, and it is quite large.

The second part of the database contains the REPFIND results tables. This part is split into two identical tables, 'match' and 'match_random'. All motif clusters having P-value less than 10^-4 ^were stored for retrieval and analysis. Clusters with P-values higher than 10^-4 ^were not included in these tables. There are two indices for both the 'match' and the 'match_random' tables. One indexes on P-value and uses the binary tree implementation (favours P-value range lookup), and one indexes on motif and uses the hash table implementation (favours value lookup). Value searches are used in the single query cases and range queries are used for the trend searches. The 'match_random' table has exactly the same characteristics and options as the 'match' table, and both tables are indexed to allow fast joining to the 'insdseq' table.

### Generation of motif data for 'match' and 'match_random' tables

REPFIND was used to identify the clustered motifs in each of the 3'-UTRS. REPFIND functions by calculating a P-value for every cluster of every repeated motif greater than 3 nucleotides long in a single 3'-UTR. It then outputs only the cluster with the lowest P-value while all other clusters of the motif are discarded. Consequently, REPFIND reveals the region of the 3' UTR that has the most significant number of a repeated motif its specific nucleotide composition [20]. REPFIND is available from . Each 3'-UTR sequence was read from the database, and collected into a single file. This entire file was input into REPFIND, which operates on each gene independently. Since long repeats are easily identified with alignment tools we included all motifs from 3–255 in length, but repeats longer than 255 were not included. Using REPFIND on a large number of genes requires a lot of computing cycles. Therefore, each organism was analyzed individually on different computers in order to parallelize the process. The data were collected into files that were easy to import into the database.

### Creation of the 3'-UTR SIRF website

A web application was developed to analyze the contents of the databases. There are two main features this website. The first is a *Single-motif search *that generates lists of genes containing an abundance of a given motif in its 3' UTR. The second is a *Trends *graphing which shows how frequently clusters of a given class of motifs occurs in the 'match' and 'match_random' tables.

#### Single-motif search

This feature provides query windows that are used to set the search parameters to describe a specific class of repeated motifs. The user provides an organism, maximum and minimum motif length, a motif or sub-motif in the IUB/IUPAC format, and a P-value cut-off. The application returns all genes containing this class of motif as a list. At the top of the list is the total number of genes identified in the 'match' and 'match_random' tables. This number is useful for providing the user a sense of whether the number of genes would be expected by chance. For example, if a search is carried out for motifs that contain "CAC", are 5–7 nucleotides long, and are found in clusters with P < 10^-6^, 298 genes are identified in the human 'match' database, whereas only 1 gene meets these search criteria in the human 'match_random' database. This suggests that most, if not all clusters of CAC-containing motifs did not arise by chance, and may therefore have been selected through evolution for a specific biological function. The user can see which of the genes when randomized produced a cluster of CAC-containing motifs meeting the search criteria by clicking the 'randomized UTRs' button. This information may be useful for some purposes.

On this same search application is a checkbox to specify "Show only best match" (Match with lowest P-Value). The default is to leave this in the checked state such that searching for TGCAC in *Xenopus laevis *returns only the TTGCAC motif for BC076786 even though clusters of three other similar motifs (ATTGCAC, TGCACT, TGCAC) exist in this same 3'-UTR (P < 10^-6^); these two additional motifs are shown if the "Show only best match" is unchecked. In addition, unchecking the "Show only best match" box causes the number of total clusters (not genes) to be given for the 'match' and 'match_random', respectively. For example, unchecking the "Show only best match" box with the same search parameters used above for CAC-containing motifs (P < 10^-6^, 5–7 mers) in human 3' UTRs yields 645 clusters in the real and 1 cluster in the randomized data set. This indicates that the real 298 3'-UTRs identified in this search contain an average of two CAC clusters each, and the gene which appears on the randomized list has only 1 cluster of a CAC-containing repeat. When the results are returned from the database, this web application outputs the genes ranked by P-value, including a brief description of each gene, and a link to the GenBank sequence entry on NCBI. The number of motifs and size of the cluster in nucleotides is also provided with the indicated P-value. Since these motifs are small, they are often also found outside the indicated cluster. However, including motifs outside the indicated cluster was determined by REPFIND to increase the P-value and such clusters of that specific motif were consequently not included in the database.

#### Trends

**Figure 2:**
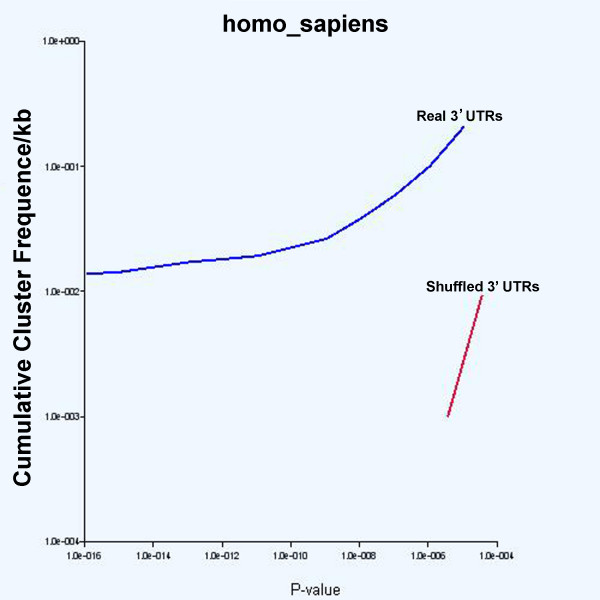
**Cumulative cluster frequencies of CAC-containing motifs in human 3'-UTRs**. *Trends *was used to determine the cumulative frequencies of clusters of 5–7 nucleotide long CAC-containing repeated motifs in the 'match' table (blue line) and 'match_random' table (red line). As can be seen, the frequencies of CAC-containing motifs with low P-values are much higher in real 3'-UTRs than they are in the shuffled ones. This type of separation is seen in all seven vertebrate species and with independently shuffled control data sets (data not shown).

The second main feature of the 3'-UTR SIRF web application (*Trends*) is used to give the user a quantitative estimate of the significance a motif with a particular P-value has in a given set of genes. It provides an on-demand graphical representation of the cumulative frequencies at which a specific class of motifs occurs in the real and "randomized" databases. This part of the application creates a graph that plots the cumulative frequency at which all clusters in all 3' UTRs with less than a specified P-value occur. To create the graph, the user inputs an organism and a motif class, and the application calculates the number of clusters that fit these parameters for both the 'match' and the 'match_random' tables. This is useful for determining whether a class of motifs is present at higher frequency in the entire set of 3'-UTRs than would be expected by chance in a shuffled dataset with identical nucleotide frequencies. For example, a search of 5–7 mer CAC-containing motifs in human genes shows that the real 3' UTRs have at least an order of magnitude more clusters meeting the search criteria than do the shuffled genes at P-values less than 10^-4 ^(Figure 2). Moreover, no clusters are found in the random set with a P-value less than 10^-7^. The cumulative frequency data plotted are also provided in tabular form below the graphical outputs to allow the user to import the data into other software programs. Finally, when REPFIND is used to analyze multiple independently shuffled data sets, *Trends *generates cumulative frequency plots that very similar varying on average by about two fold (data not shown).

#### Query by NCBI Accession

This feature provided on the 3'-UTR SIRF website displays all motifs that are associated with a specific gene. They are ordered by P-value. Because of the low P-values characteristic of long repeats, the list is often dominated by motifs greater than 50 nucleotides in length if such motifs exist in the sequence.

#### Multi-Motif Search

Another component of 3'-UTR SIRF is the "Multi-Motif Search" tool which can be used to identify genes containing two distinct repeated motifs of interest. For example, a search for human 3'-UTRs containing two specific 3 mers (CAC, CAG) with P < 10^-4^, reveals 717 genes that contain significant numbers of both motifs in their 3' UTRs. No genes fitting these criteria were identified in the shuffled dataset. This search engine is highly specific in that it requires a perfect match to the input motifs and does not yet have the ability to search for combinations of general motifs classes.

## Utility and discussion

The utility of any new Bioinformatics tool such as 3'-UTR SIRF resides in its ability to make valid predictions that lead to progress in our understanding of a particular biological process. As mentioned above, the *cis*-elements that specify cytoplasmic mRNA localization in vertebrates often contain many short repeated RNA motifs that are required for their function [13,14,16,17,20-22]. To identify new RNA localization elements in human genes we used two computational strategies. The first strategy involved utilization of 3'-UTR SIRF to identify human genes that contain significant clusters of short CAC-containing motifs characteristic of many RNAs that become localized to the vegetal pole of *Xenopus *oocytes [13,14,16,17,20-22]. In the second approach, we used REPFIND to analyze the 3' UTRs of mRNAs that are known to localize to the dendrites of mammalian neurons. We discovered that CAG-containing motifs are abundant in many of these transcripts and used 3' UTR SIRF to identify additional transcripts with CAG-rich 3' UTRs. We then tested whether these mRNAs are also localized in mammalian neurons. Our results suggest that both approaches are viable for computationally predicting localized mRNAs from mRNA databases.

### Identification of functional CAC-rich RNA localization elements in human genes

In our first approach to identify human RNA localization elements, we used 3'-UTR SIRF to search for mRNAs that contain clusters of CAC-containing motifs with low P-values in their 3'-UTR. This was done for two reasons. First, mRNAs localized in mammalian neurons, such as *β-actin *and *RhoA*, contain clusters of CAC-containing motifs similar to those required for RNA localization in *Xenopus *oocytes [20] (data not shown). Secondly, a computational search for 3'-UTRs containing significant clusters of CAC-containing motifs in *Xenopus *resulted in the reliable prediction of new localized mRNAs in oocytes [9,20]. To identify new localized mRNAs in humans, we used 3'-UTR SIRF to search for mRNAs with repeated CAC-containing motifs 5–7 nucleotides long with P-values less than 10^-6^. *Trends *revealed a large separation between the cumulative frequencies of these CAC-rich motif clusters in the human 3'-UTRs and their shuffled counterparts (Figure 2), and this degree of separation is observed in all vertebrate species in the database (data not shown). In addition, this difference between real and shuffled is maintained when multiple independently shuffled databases are used as a control data set; the cumulative frequencies of repeats found in independently shuffled databases vary only by about two fold (data not shown). As mentioned above these search parameters (CAC 5–7 mers, P < 10^-6^) yield 298 human genes with only one in the random set.

**Figure 3:**
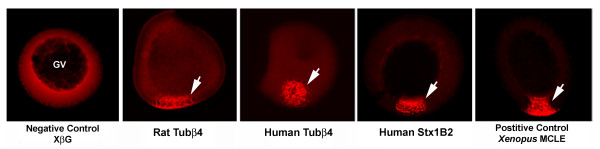
**Localization of the Rat and Human *Tubβ4 *3'-UTRs in *Xenopus *oocytes**. The 3'-UTR of rat or human *Tubβ4 *(Acc. # 82522352 and BC013683, respectively), and human *Stx1B2 *(Acc. # BC062298) were synthesized and labelled *in vitro *with Alexa-Fluor-546-UTP. These fluorescently labelled RNAs were then microinjected into stage II *Xenopus *oocytes. All three RNAs localize to the vegetal pole, which is oriented downwards in all panels. A fragment of the *Xenopus *β-*globin *gene (*XβG*) was used as a negative control for localization, whereas the mitochondrial cloud RNA localization element from the *Xenopus Xcat-2 *mRNA (MCLE) was used as a positive control. Note that the extent of *Stx1B2 *localization is higher than that of either *Tubβ4 *RNA. Arrows depict the localized RNA towards the vegetal pole and GV indicates the germinal vesicle (nucleus) in these cells which are ~300 μm diameter.

**Figure 4:**
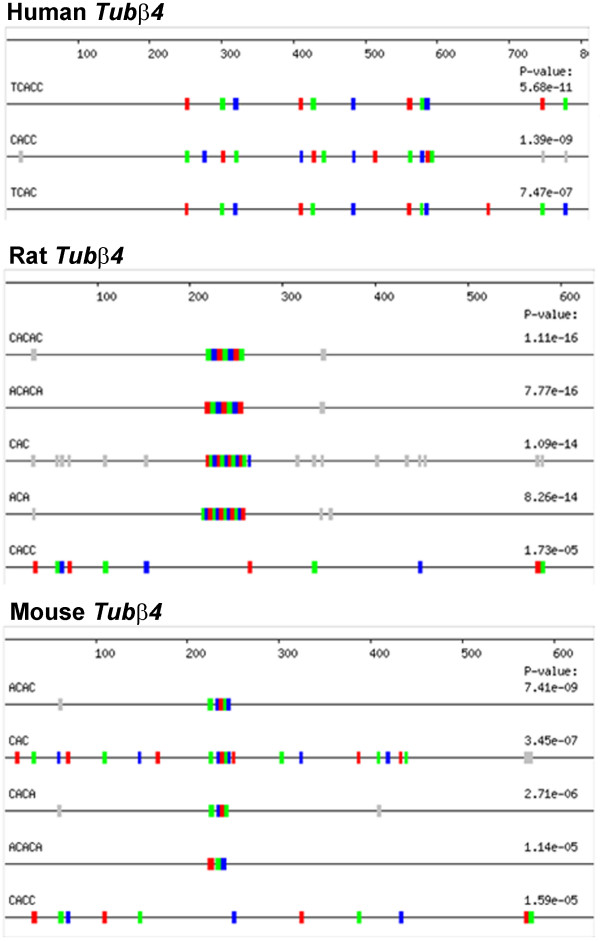
**Mouse, Rat, and Human *Tubβ4 *3'-UTRs all have an abundance of CAC-containing motifs**. Even though the human *Tubβ4 *3'UTR has little sequence similarity when it is aligned with the mouse or rat orthologs, all three genes are shown to have a highly significant number of CAC motifs when individually assessed by REPFIND. For the rat and mouse sequences, REPFIND was performed without filtering low complexity regions and the human background was used. The accession number for the mouse *Tubβ4*gene is BC054831. Motifs depicted in grey would have yielded higher (less significant) P-values, and therefore were not used to generate the P-values shown.

Since proteins, such as Staufen [5-7] and Kinesin 2 [8,9] mediate mRNA localization in *Xenopus *oocytes and mammalian neurons, we tested whether two genes identified on the list of 298 CAC-rich human 3'-UTRs possess RNA localization activity using the *Xenopus *oocyte system. These two genes, *Tubulin β4 *(*Tubβ4*) and *Syntaxin 1B2 *(*Stx1B2*), were chosen because they are known to be expressed in mammalian neurons and, therefore, may localize if injected into *Xenopus *oocytes. *Tubβ4*, which encodes an isoform of β-Tubulin that is specifically expressed in neurons [23], was fluorescently labelled *in vitro *by incorporation of Alexa-Fluor-546-UTP and injected into stage II *Xenopus *oocytes as previously described [22]. Two standard controls were used for localization. The first is a fragment of the *Xenopus β*-*globin *(*XβG*) gene that does not localize in oocytes. The other is the mitochondrial cloud localization element (MCLE) of the *Xenopus Xcat-2 *mRNA that recruits Kinesin II [9] and localizes extremely efficiently to the vegetal cortex of stage II oocytes [22,24]. The results (Figure 3) show that the human *Tubβ4 *3'-UTR localizes well in *Xenopus *oocytes. *Tubβ4 *appears in an identical 3'-UTR SIRF search of mouse sequences, but not in a search of rat genes because the rat *Tubβ4 *gene is not included in the MGC database. However, we identified the rat *Tubβ4 *3'-UTR encoded by an EST, and it is also enriched in CAC-containing motifs identified by REPFIND (Figure 4). Therefore, we cloned the rat *Tubβ4 *3'-UTR and tested it for RNA localization activity using the *Xenopus *oocyte assay. This experiment shows that the rat *Tubβ4 *3'-UTR also localizes in *Xenopus *oocytes (Figure 3). These results indicate that the *Tubβ4 *mRNA localization element is evolutionarily conserved in mammals, and it likely functions in establishing polarized expression of *β*-Tubulin for specialized cellular functions. Interestingly, while REPFIND reveals common CAC-containing motifs in the *Tubβ4 *3'-UTR from distinct mammalian species (Figure 4), alignment tools fail to do so (data not show). This underscores the importance and utility of the REPFIND algorithm for identifying short RNA motifs with evolutionarily conserved regulatory functions.

Human *Stx1B2 *is the second CAC-rich 3'-UTR that we tested for localization in *Xenopus *oocytes. This gene encodes a tSNARE that is thought to be important for vesicle docking and the release of neurotransmitters that contribute to memory functions of the hippocampus [25]. In addition, Stx1B2 protein localizes to axons and synaptic terminals of motor neurons [26]. When the human STX1B2 3'-UTR is fluorescently labelled and injected into early stage *Xenopus *oocytes it also becomes localized to the vegetal pole. In fact, the extent of its localization, characterized by the amount of fluorescent signal in the vegetal region compared to the surrounding cytoplasm, is more robust than that of the *Tubβ4 *3'-UTR (Figure 3).

While CAC-containing motifs have been shown to be required for localization of several mRNAs in *Xenopus *oocytes, the motifs alone are not sufficient for RNA localization [27] suggesting that the sequence context of CAC motifs is critical for the functional integrity of these localization elements. Since *Tubβ4 *and *Stx1B2 *have an abundance of CAC motifs and localize in *Xenopus *oocytes (Figure 3), we conclude that both the human *Tubβ4 *and *Stx1B2 *genes contain a *bona fide *CAC-rich mRNA localization element in their 3'-UTRs. This functional analysis of two human CAC-rich 3' UTRs in *Xenopus *oocytes demonstrates for the first time that RNA localization signals in non-coding regions of mRNAs can be identified in human genes using computational methods. Moreover, since two CAC-rich 3' UTRs, of two tested, have localization activity, these results suggest that the reliability at which 3' UTR SIRF predicts functional RNA localization signals may be quite high. However, more genes need to be tested to determine the success rate of predicting CAC-rich RNA localization elements in the 3'-UTR SIRF database.

### Identification of abundant CAG-containing motifs as another feature of mRNAs localized to the neurites of mammalian neurons

**Figure 5:**
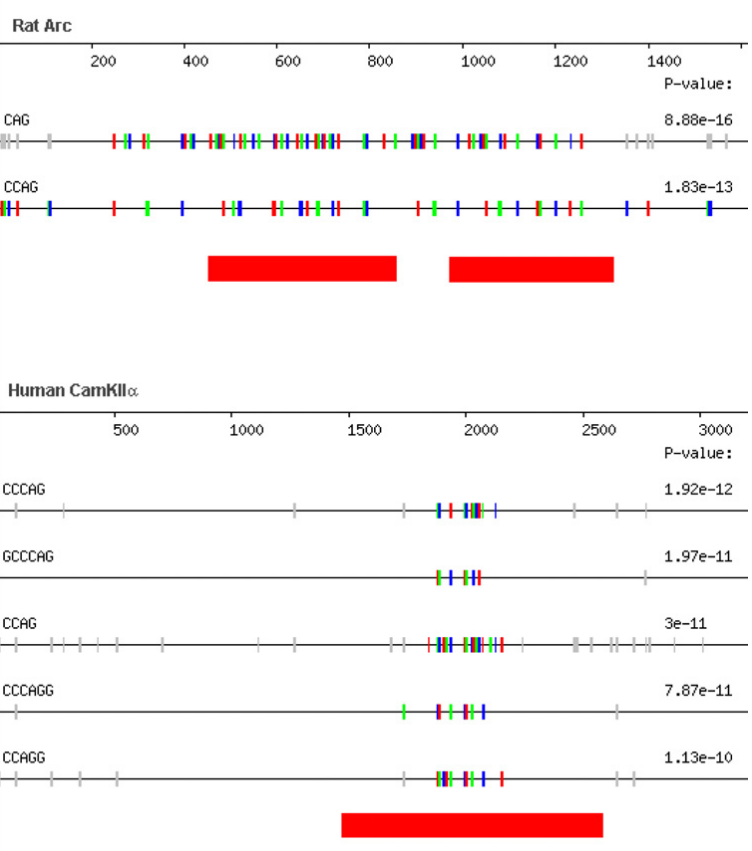
**REPFIND analysis of dendritic mRNAs *CamKIIα *and *Arc***. The 3'-UTR of rat *Arc *(Acc. #NM_019361) and human *CamKIIα *(Acc. #BC012321) were analyzed for all repeats. As can be seen, CAG or CAG-containing motifs comprise the top scoring cluster for each 3'-UTR. Motifs depicted as vertical small colored bars indicate the cluster with the most significant P-value. The red bars below each 3'-UTR represent RNA sequences that have dendritic RNA localization activity and were mapped in previous studies using reporter assays [28, 29].

Many mRNAs have been shown to localize to the dendrites of mammalian neurons. However, common *cis*-elements have not yet been identified in this class of localized mRNA [28-33]. In a second approach to identify novel mRNA localization signals in human genes, we used REPFIND  to examine the 3'-UTRs of several previously characterized dendritic mRNAs. We found that CAG or CAG-containing motifs were the most abundant motif in many of these 3'-UTRs. Moreover, several of the CAG clusters correspond to regions of the 3'-UTRs that have been shown to have dendritic RNA localization activity in previous studies. REPFIND outputs of two well-characterized 3' UTRs (*CamKIIα *and *Arc*) are shown in Figure 5. As can be seen, CAG itself is the best scoring repeat in the rat *Arc *3'-UTR (P ~10^-15^), whereas, CCCAG is the most significant repeat in the human *CamKIIα *3'-UTR. In addition, these clusters overlap with previously identified segments of these 3' UTRs that have been shown experimentally to have RNA localization activity [28,29] (Figure 5). This suggests that CAG rich 3'-UTRs may be a common feature of at least some 3'-UTRs that specify localization to dendrites.

To identify other genes that contain an unusually high number of CAG motifs in their 3'-UTRs, we performed a *Single-motif search *with parameters identical to those used to identify CAC-rich genes. The result was 749 human genes containing clusters of CAG-containing motifs 5–7 nucleotides long (P < 10^-6^), with only two genes being present in the shuffled data set. Moreover, *Trends *showed a large number of CAG-containing clusters in the real, but not the shuffled 3'-UTR dataset (data not shown).

To determine if any of the candidate 749 mRNAs are indeed localized to the neurites of neurons, *in situ *hybridization was performed on primary rat hippocampal neurons using a procedure that was capable of identifying a microRNA in dendrites [34]. The target genes to be analyzed were *Sec61α *and *Syntaxin 1A *(*Stx1A*). These two genes where chosen in part because whole mount *in situ *hybridization of entire mouse brains indicates they are expressed in the hippocampus (Allen Brain Atlas), and therefore, are likely to also be expressed in cultured hippocampal neurons. However, since endogenous transcripts were to be assessed, the rat 3'-UTRs were identified in the EST database available at NCBI, cloned, and used to make digoxigenin labelled antisense RNA probes for detection of the endogenous rat orthologs. Rat orthologs were cloned by using tBLASTN to identify rat ESTs that show perfect or nearly perfect amino acid identity with a C-terminal region of the orthologous human open reading frame. Each 3' UTR identified in this way was amplified from rat genomic DNA using the polymerase chain reaction. Amplified products were cloned into a T7 promoter-containing transcription vector such that antisense transcripts could be synthesized *in vitro*. All plasmid constructs were verified by DNA sequencing. Antisense probes were also generated for two rat CAC-rich mRNAs, *Tubβ4 *and *Styntaxin1B2 *(*Stx1B2*). As negative controls for localization, two different antisense RNA probes were used. One is complementary to the rat ortholog of human *Syntaxin5 *(*Stx5*), and the other is complimentary to the rat *αTublin3A *(*αTub*) gene which has been used previously as a negative control for RNA localization studies in mammalian neurons [35]. Neither of these transcripts has an abundance of CAC or CAG motifs in their 3'-UTRs. All probes were designed to be complementary to 3' UTRs to reduce the possibility of cross hybridization to homologous transcripts encoding other protein family members. The sequences of primers used for cloning these DNA fragments are shown in Table 1.

**Table 1:** DNA Oligonucleotides used for PCR amplification and cloning of 3' UTRs with restriction sites in bold text.

**Gene Designation**	**Organism**	**Accession #**	**5' Primer**	**3' Primer**	**PCR Product Size**
*Syntaxin 1a*	Rat	NM_053788	CTGCTGGTGT**AAGCTT**AGCACCCAGTACCCCTCTTT	ACCTTTGGTG**GAATTC**TAAAGGGAAGTGGCCATGAG	598
*Sec61α*	Rat	NM_199256	CCAGAACTGC**AAGCTT**AGGGTGCTCTTACTGCTGGA	GAAGACAGAG**GAATTC**ACCACAGGACCTCCCTTTCT	496
*Syntaxin 1B2*	Rat	NM_012700	TGGATCCCCC**AAGCTT**TTGCCGCACATAGATAGCAG	TTTGTTCTAC**GAATTC**AAAGATGTGTGGCATGGTCA	468
*Syntaxin 1B2*	Human	BC062298	TCCAGAGGCC**GAATTC**ACCCTTCTCTCTCCCAGACC	TAAGCCACCC**AAGCTT**CAGTGGCTTTGTTGCTGTTG	573
*Syntaxin 5*	Rat	NM_031704	CCATGGAGGG**AAGCTT**ACCCTTCTGGAAGGACAGGT	CCCCCCACCT**GAATTC**GTGAGGAGAAGGTGGCAGTC	404
*Tubα3*	Rat	CH473964	GGGCTGCAGG**AAGCTT**GCTTCCTCATCTTCCACAGC	TTTGTGGATT**TCTAGA**CTGGATGGTACGCTTGGTTT	626
*CamKIIα*	Rat	NW_047514	AGCAAGCCCGT**GAATTC**GCACACCACCATCCTGAAC	GCGCCCTCCG**GTCGAC**CCCAGATCTGTGGAAGTGGA	184
*Tubβ4*	Rat	82522352	GAGCAATATG**GAATTC**AACGACCTGGTGTCCGAGTA	TGAGTCATTGC**GTCGAC**TTTATTGATGGAGGGTCTGC	750
*Tubβ4*	Human	BC013683	AGGCTGCTCC**GAATTC**CATCGCTTCCCACCTGTC	ACAAGGCCTG**AAGCTT**TTCTCTCCCAGATAAGCTAAGGTC	710

**Figure 6:**
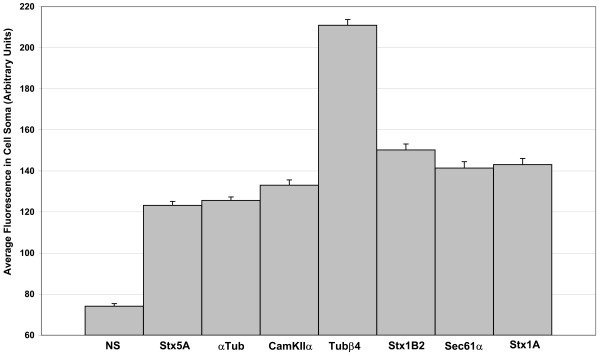
**Verification that *in situ *hybridization labels specific endogenous transcripts**. To verify that the *in situ *hybridization detects specific endogenous transcripts, average labelling of the cell bodies was quantified from more than 30 cells for each probe and compared to a non-specific (NS) RNA probe. Error bars show standard error of the means. The Student t-Test shows all probes produce much stronger labelling in the cell body than observed with the non-specific probe (P < 0.0001) which was not different than labelling seen when the RNA probe was completely omitted from an otherwise identical protocol (data not shown). The *in situ *procedure used for these studies was adapted from a previous study [34]and involved hybridization of a digoxigenin-labelled RNA probe, labelling of this probe with an anti-digoxigenin fluorescein-conjugated antibody followed by amplification with a secondary Cy3-conjugated mouse monoclonal anti-fluorescein antibody. All images were acquired on a Zeiss LSM 510 confocal laser scanning microscope.

Previous work has shown that mRNAs localized to dendrites or axons also exist at high levels in the cell body. We took advantage of this to optimize our *in situ *hybridization protocol. We optimized blocking conditions and hybridization temperature (60°C) such that there was little difference in cell body fluorescence between cells incubated without an RNA probe and cells incubated with a non-specific RNA probe. At the same time, such conditions must also result in a robust fluorescent signal with antisense probes to mRNAs known to be expressed in neurons. One such mRNA is *CamKIIα *which has become a standard control for mRNA localization studies. To establish that each RNA probe specifically labels its complementary endogenous target mRNA, we quantified fluorescence in cell bodies from many cells hybridized to each probe. The signal was compared to cells incubated with either no RNA probe or a non-specific (NS) RNA probe. Each probe results in fluorescent signals in the cell body that are similar to or greater than that observed with *CamKIIα *(Figure 6). These data show that the *in situ *procedure specifically detects distinct mRNAs in these cultured rat hippocampal neurons, and that each gene is expressed at different levels in the cell body with *Tubβ4 *being most highly expressed of all genes tested.

**Figure 7:**
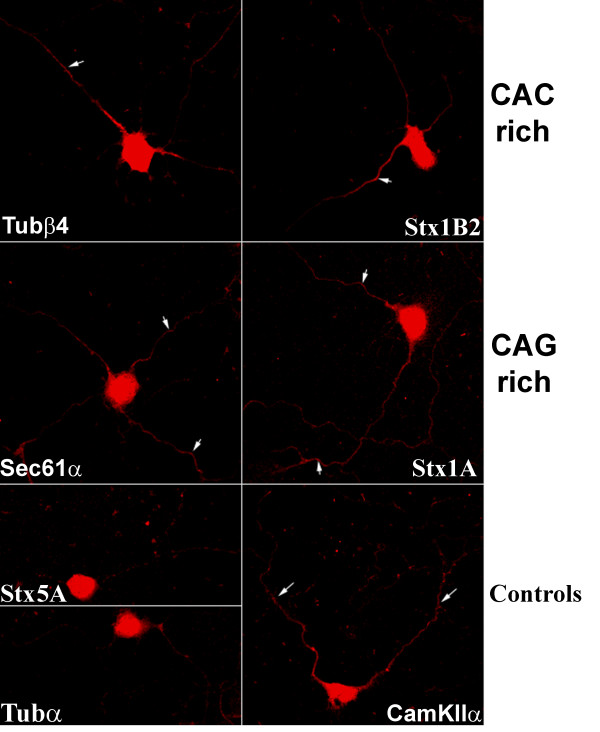
**Endogenous CAC and CAG rich mRNAs are localized to distal processes in mammalian neurons**. *In situ *hybridization was used to reveal the subcellular distribution of each mRNA in rat hippocampal neurons that had been cultured for 8 days after plating. *Stx5 *was used as a negative control for localization since it has no repeats and resides exclusively in the cell body. *CamKIIα *was used as a positive control for localization since it is well known to localize well to distal processes. White arrows show labelling in distal processes. All images were collected at identical laser settings using confocal microscopy and all images were processed together as a montage image to enhance contrast. In addition all cells came from the same experiment and each cell has multiple processes in the focal plane, but often a single process is preferentially labelled. The identity of processes as either axons or dendrites is not yet known. Specific mRNAs were detected in distal processes with both CAC-rich mRNAs (*Tubβ4 *and *Syn1B2*) and both CAG-rich mRNAs (*Syn1A *and *Sec61α*) that were identified with 3'-UTR SIRF. The cell bodies in these images are approximately 15 μm in diameter.

Using the above conditions for *in situ *hybridization we were able to detect reliably fluorescence in the neurites of cells hybridized to the *CamKIIα *probe in cultured rat hippocampal neurons. Fluorescence above background was not observed in the neurites of cells hybridized to an antisense probe complementary to the negative controls, *αTub *or *Stx5*, both of which primarily label the cell body (Figure 7). Remarkably, both mRNAs containing CAC-rich 3'-UTRs (*Tubβ4 *and *Stx1B2*) and both containing CAG-rich 3'-UTRs (*Sec61α *and *Stx1A*) were also detected in distal processes away from the cell body (Figure 7).

**Figure 8:**
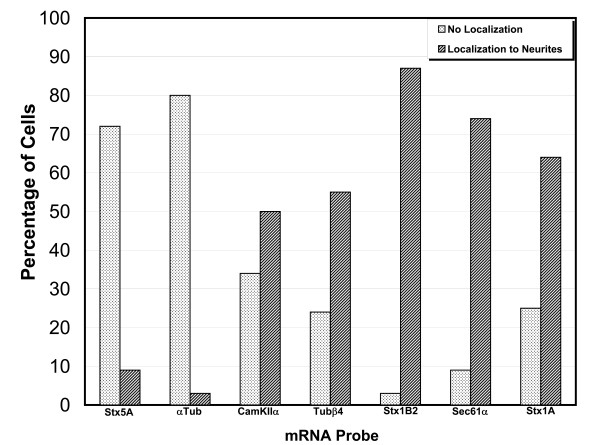
**Semi-quantitative analysis of the localization of endogenous mRNAs**. To estimate the extent of localization of each endogenous mRNA, images were collected from 30–40 cells using identical laser settings from the same experiment shown in Figure 7. All raw images were assembled into a montage and a threshold was applied to help identify mRNA labelled in distal processes. A cell was considered to be positive for localization if mRNA could be detected in a process greater than 40 μm away from the cell body. If no signal could be detected greater than 10 μm away from the cell body the cell was considered to be negative for mRNA localization. About 15–30 percent of all cells showed some signal in processes 10–40 μm away from the cell body. These cells were excluded from the graph since they added little information to this analysis.

To provide a semi-quantitative assessment of these localization patterns, we analyzed the distribution of each mRNA in many neurons and counted the number of cells that show labelling in neurites at least 40 μm away from the cell body for each RNA probe (Figure 8). This analysis showed that greater than 50 percent of cells hybridized with antisense probes specific for *CamKIIα*, *Stx 1A*, *Sec61α*, *Stx 1B2 *and *Tubβ4 *localized to distal neurites with several cells showing labelling in neurites over 100 μm away from the cell body. In contrast, only ~10 percent of cells hybridized with probes specific for *α-tubuli*n or *Stx5 *showed localization to neurites (Figure 8).

One question emerging from this study is how many mRNAs encoded by the genome contain mRNA localization signals? Interestingly, the percentages of CAC-rich (Table 2) and CAG-rich (Table 3) genes identified by 3'-UTR SIRF are similar in all vertebrates tested with randomized 3'-UTR data sets showing 10 to 100 fold fewer genes depending on P-values. Moreover, ~10 percent of genes in the real 3'-UTR database contain either a CAC-rich (Table 2) and/or a CAG-rich (Table 3) region of their 3' UTR (P < 10^-4^). This P-value is within the range of previously characterized CAC-rich RNA localization elements [20], and the ~10 percent estimate of genes containing localization signals is similar to experimental estimates obtained in mammalian neurons [36-38] and *Drosophila *oocytes [39]. Therefore, while further work is required to determine the reliability with which 3'-UTR SIRF predicts mRNA localization elements, this computational analysis suggests that short CAC- and CAG-containing motifs may be part of a widely utilized genetic code for specifying polarized patterns of gene expression in human cells. Since CAC-rich mRNAs, such as *Stx1B2 *[26], *rho *[40]and *β-actin *[41], encode proteins that are targeted to axons, and CAG-rich RNAs, such as *CamKIIα *and *Arc *are localize to dendrites [42], it is tempting to speculate that CAC-rich RNA localization elements may provide a general signal for preferentially targeting mRNAs and their encoded proteins to axons, whereas CAG-rich RNA localization elements may provide a general signal for dendritic targeting. However, double labelling experiments with compartment-specific markers will be required to test this directly. Finally, it should be emphasized that this is the first study to demonstrate the feasibility of using a computational analysis of non-coding mRNA sequences to predict functional mRNA localization signals in human and other mammalian mRNAs on a genome-wide scale. Further utilization of this computational approach may allow the identification of additional signals in 3' untranslated regions that regulate other post transcriptional aspects of gene expression.

**Table 2:** Percentage of CAC-rich 3'-UTRs in vertebrate genes.

**CAC 5–7 mer**	**Zebrafish **(7965)		**Frog **(8406)		**Mouse **(16,594)		**Human **(20,924)	
**P < 10**^-7^	43	0	64	0	125	1	149	0
	**0.5%**		**0.8%**		**0.7%**		**0.7%**	
**P < 10**^-6^	85	0	107	0	278	5	298	1
	**1.1%**		**1.3%**		**1.7%**		**1.4%**	
**P < 10**^-5^	178	3	212	15	654	36	779	16
	**2.2%**		**2.5%**		**4.0%**		**3.7%**	
**P < 10**^-4^	496	49	593	105	1723	263	1895	221
	**6.2%**		**7.1%**		**10.4%**		**9.1%**	

Numbers in parentheses to the right of each species indicate the total number of genes analyzed. Numbers above percentages indicate the number of real (left) and random (right) 3' UTRs that contain at least one cluster of CAC motifs with the indicated P-value.

**Table 3:** Percentage of CAG-rich 3'-UTRs in vertebrate genes.

**CAG 5–7 mer**	**Zebrafish **(7965)		**Frog **(8406)		**Mouse **(16,594)		**Human **(20,924)	
**P < 10**^-7^	70	0	95	0	219	0	392	1
	**0.9%**		**1.1%**		**1.3%**		**1.9%**	
**P < 10**^-6^	136	0	163	2	470	3	749	2
	**1.7%**		**1.9%**		**2.8%**		**3.6%**	
**P < 10**^-5^	265	8	375	15	1149	23	1520	22
	**3.3%**		**4.5%**		**6.9%**		**7.3%**	
**P < 10**^-4^	693	43	949	95	2769	239	3215	236
	**8.7%**		**11.3%**		**16.7%**		**15.4%**	

Numbers in parentheses to the right of each species indicate the total number of genes analyzed. Numbers above percentages indicate the numbe of real (left) and random (right) 3' UTRs that contain at least one cluster of CAG motifs with the indicated P-value.

## Conclusion

In this work we used the REPFIND algorithm [20] to identify all repeated motifs in thousands of 3'-UTRs from seven vertebrate sequences available in the Mammalian Gene Collection at NCBI. These motifs were stored in the 3'-UTR SIRF database, and a search tool was developed to extract individual sequences that contain an abundance of any user-defined repeat. Since previous work has shown that mRNA localization signals in *Xenopus *are often enriched in short CAC-containing motifs we searched for human and other mammalian genes that contain clusters of CAC motifs in their 3'-UTRs. This computational analysis suggests that up to 10 percent of human genes may contain CAC-rich RNA localization signals, and two of these genes, *Tubβ4 *and *Stx1B2*, were experimentally validated to contain functional RNA localization sequences. In addition, we discovered that several RNAs shown previously to localize to the dendrites of mammalian neurons are enriched in short CAG-containing motifs. *In situ *hybridization was used to validated that two new candidate CAG-rich mRNAs identified with 3'-UTR SIRF are also localized to neurites of cultured neurons, whereas control RNAs lacking repeated motifs remain in the cell body. Together these studies suggest that short reiterated RNA sequence motifs may comprise part of a widespread genetic signal present in thousands of genes for generating polarized patterns of gene expression in mammalian cells. Further work will be required to test this idea directly and to determine whether CAC motifs, CAG motifs, and/or higher order RNA structure may specify whether distinct mRNAs become targeted preferentially to axons or dendrites in mammalian neurons.

## Availability and requirements

3'-UTR SIRF is freely available to those in academic settings [43]. However, those wishing to use it for commercial purposes must contact JOD or Boston University prior to doing so.

## Authors' contributions

BBK and IL wrote and designed all software, in addition to constructing the motif database. This project was initiated as part a bioinformatics database course taught by GB at Boston University. The molecular work and *in situ *hybridization experiments were performed by JJV and MTF. BH performed the RNA localization assays in *Xenopus *oocytes. LAJ and HM provided rat hippocampal neurons and aided in establishing the *in situ *hybridization protocol. JOD conceived the project, selected the genes to be tested with help from MTF, wrote the paper, and generally supervised all aspects of the work.
